# Multiple Endocrine Neoplasia Type 1 with Concomitant Existence of Malignant Insulinoma: A Rare Finding

**DOI:** 10.1155/2021/8842667

**Published:** 2021-07-28

**Authors:** Randhir Sagar Yadav, Ashik Pokharel, Deepshikha Gaire, Shumneva Shrestha, Ashbita Pokharel, Sumita Pradhan, Prasan Bir Singh Kansakar

**Affiliations:** ^1^Maharajgunj Medical Campus, Institute of Medicine, Tribhuvan University, Kathmandu, Nepal; ^2^Department of Gastrointestinal and General Surgery, Tribhuvan University Teaching Hospital, Kathmandu, Nepal; ^3^Department of Pathology, Tribhuvan University Teaching Hospital, Kathmandu, Nepal

## Abstract

Multiple endocrine neoplasia type 1 (MEN1) is a rare syndrome of autosomal dominant inheritance defined by co-occurrence of two or more tumors originating from the parathyroid gland, pancreatic islet cells, and/or anterior pituitary. Insulinoma which has an incidence of 0.4% is a rare pancreatic neuroendocrine tumor. Malignant insulinoma is extremely rare, while primary hyperparathyroidism is a common occurrence in MEN1. We present a case of MEN1 syndrome with 2.6 cm insulinoma in the pancreatic head and parathyroid adenoma in a 56-year-old female who presented with symptoms suggestive of hypoglycemia like multiple episodes of loss of consciousness for four years. Classical pancreaticoduodenectomy was carried out, and the postoperative period was uneventful. Later, subtotal parathyroidectomy was performed, which showed parathyroid adenoma. Patients presenting with features of hypoglycemia should be vigilantly assessed for the presence of a sinister pathology.

## 1. Background

Multiple endocrine neoplasia type 1 (MEN1) is an autosomal dominant disorder characterized primarily by two or three principal MEN1-related endocrine tumors originating from parathyroid glands, endocrine gastroenteropancreatic (GEP) tract, and anterior pituitary [1]. Primary hyperparathyroidism is found in 90% of patients with MEN1 syndrome [1], whereas only 5–10% insulinomas present as a constituent of MEN1 [2]. Most of the insulinomas are benign; very rarely, they are malignant [3]. Whipple's triad along with supervised fasting serum insulin, C-peptide, and proinsulin thresholds/cutoffs along with imaging studies are vital in diagnosis and localization of mass [4]. Surgical resection is the mainstay of treatment of insulinoma [4] and parathyroid adenoma [1].

## 2. Case Presentation

A 56-year-old lady presented to us with a history of multiple episodes of loss of consciousness mostly on empty stomach for 4 years with 15–20 fainting attacks per year. She was hospitalized multiple times at local health facilities for hypoglycemia, and she was managed symptomatically. Except for some other neuroglycopenic and adrenergic symptoms of hypoglycemia, there were no other systemic complaints, findings, and significant medical or surgical history. Her family history revealed that her two brothers and two sons died of unknown diseases at a young age (20–37 years). Due to financial constraints, they were not able to undergo any medical investigation or treatment. The systemic examination was normal.

**Figure 1:**
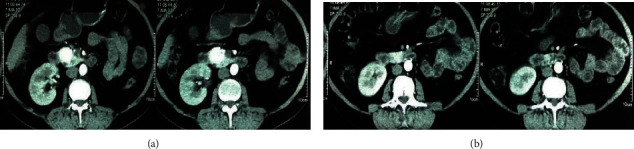
Hyperattenuating lesion in the anterior plane of the head of the pancreas (2.4 cm × 2.3 cm × 2.1 cm), bilateral adrenal hyperplasia, and nonvisualization of the left kidney.

**Figure 2:**
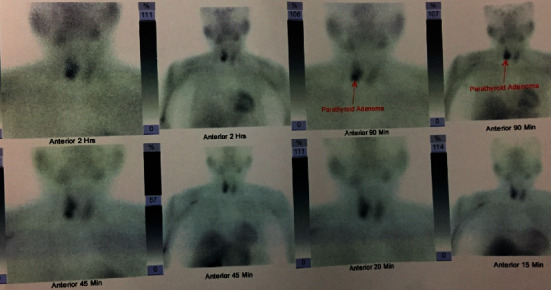
^99m^Tc MIBI scan showing right inferior parathyroid adenoma.

In the emergency room, Whipple's triad was seen (frequent episodes of hypoglycemia with a blood sugar of 34 mg/dl (normal 70–110 mg/dl) along with neuroglycopenic symptoms relieved on intravenous dextrose). The basal insulin and C-peptide were 39.3 IU/ml (normal 2.5–25.0 uIU/ml) and 4.85 ng/ml (normal 1.5–5.0 ng/m), respectively. The 72-hour supervised fasting was abandoned at 6 hours due to the development of sweating and dizziness (blood sugar 42 mg/dl). The fasting insulin (63.8 IU/ml) and C-peptide (6.82 ng/ml) were not suppressed suggesting insulinoma. She also had hypercalcemia (serum calcium 11.6 mg/dl; normal 8.3–10.3 mg/dl) with an increased parathyroid hormone (PTH) (344 pg/ml; normal < 53.5 pg/ml). Serum prolactin, thyroid stimulating hormone (TSH) and other hormonal and routine biochemical investigations were within the normal range. With suspicion of insulinoma and parathyroid adenoma, further imaging studies were performed to localize the lesion. Ultrasound of the abdomen showed a hypoechoic lesion in the pancreatic head (2.6 cm × 2.8 cm) while the left kidney was not visualized. Contrast-enhanced computed tomography (CECT) abdomen showed a hyperattenuating lesion in the pancreatic head (2.4 cm × 2.3 cm × 2.1 cm) communicating with the main pancreatic duct and bilateral adrenal hyperplasia (Figure 1). The lesion being situated in the pancreatic head did not reveal any abnormality on the upper GI endoscopy, while taking a biopsy was not possible. Esophagogastroduodenal scope-guided biopsy would have helped, but it was not available. Ultrasound of the neck showed a nodular lesion in the right lobe of the thyroid gland. Likewise, technetium-99m labeled hexakis-methoxyisobutyl isonitrile (^99m^Tc MIBI) scan showed right inferior parathyroid adenoma (Figure 2). Brain magnetic resonance imaging (MRI) was normal.

**Figure 3:**
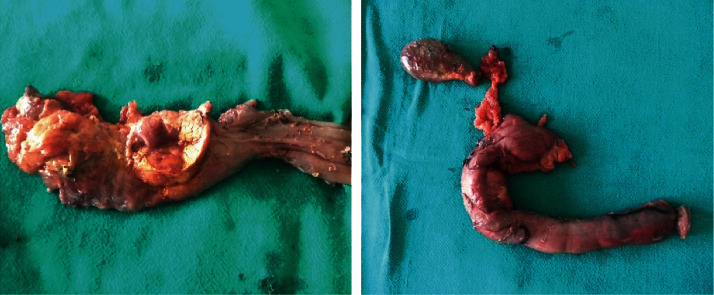
Postoperative specimen.

**Figure 4:**
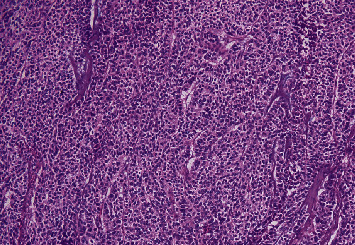
Well-differentiated neuroendocrine tumor; tumor cells arranged in trabeculae, cords, and lobules with vascular septa. These cells show mild nuclear atypia, fine stippled chromatin, inconspicuous nucleoli, and moderate amount of eosinophilic granular cytoplasm. Mitosis is infrequent (H&E X100).

Based on the clinical and investigation analyses, a diagnosis of MEN1 syndrome with insulinoma of the head of the pancreas and parathyroid adenoma was made. Classical pancreaticoduodenectomy (Whipple's procedure) was performed which showed 3 cm × 2 cm fleshy mass present in the pancreatic head (Figure 3) with no ascites, the pancreas was soft, and common bile duct measured about 12 mm. The pancreatic duct traversed through the tumor, so pancreatic resection was unavoidable. Her postoperative period was uneventful. Postoperative blood sugar, insulin, and C-peptide were 127 mg/dl, 8.9 IU/ml, and 2.28 ng/ml, respectively. Histopathological examination (HPE) revealed a well-differentiated neuroendocrine tumor (Figure 4) with a maximum tumor dimension of about 2.6 cm with lymph node metastasis. However, lymphovascular and perineural invasions were not identified. One out of seven lymph nodes resected was positive for tumor with TNM stage pT2N1. Due to the paucity of resources, immunohistochemical studies and positron emission tomography (PET) scan could not be performed. A year later, she underwent right hemithyroidectomy with excision of the right superior and inferior parathyroid glands at another center. The HPE showed infrathyroid parathyroid adenoma with nodular goiter with a hyperplastic nodule. Her biochemical parameters including blood glucose and PTH were within the normal range on regular follow-up visits, and she has been doing well clinically, and she was satisfied with the treatment and its response.

## 3. Discussion

The majority of insulinomas are sporadic, and only 5–10% insulinomas present as a constituent of MEN1 [2]. Most insulinomas unlike other pancreatic islet cell tumors (gastrinoma and VIPoma) are benign [3, 5] solitary [5]. The majority of insulinomas are less than 2 cm. Malignant insulinomas are rare. Thus, among reported cases of insulinoma, only 5–12% of cases were found malignant [3, 6–8].

The clinical presentations of insulinoma vary. Hypoglycemia is the most common presentation. Hypoglycemia manifests as neuroglycopenic as well as adrenergic symptoms. Neuroglycopenic patients present with neurological as well as psychiatric manifestations such as confusion, blurring of vision, seizure, agitation, and behavioral changes [9, 10]. Our patient also had multiple episodes of loss of consciousness due to hypoglycemia along with some neuroglycopenic and adrenergic symptoms.

Diagnosis of MEN1-associated lesions includes biochemical and hormonal evaluation, endoscopic, nuclear medicine, or other imaging studies. Moreover, clinical diagnostic criteria for MEN1 syndrome include the presence of two out of three tumors that are parathyroid, pituitary, or GEP tract tumors [1]. Whipple's triad is the classic approach of diagnosing an insulinoma while a supervised 72-hour fasting test for measurement of plasma glucose, insulin, C-peptide, and proinsulin during the onset of hypoglycemic symptoms remains the gold standard for diagnosis of insulinoma. With the availability of proinsulin assay, proinsulin cutoffs have emerged as a diagnostic tool. There is some consensus on the thresholds/cutoffs for insulin, C-peptide, and proinsulin to diagnose insulinoma [4]. Moreover, Hirshberg et al. has recommended a 48-hour fast instead of 72-hour fast [6]. Upon establishing a diagnosis, noninvasive diagnostic investigations such as transabdominal ultrasonography, computed tomography (CT), and MRI help in localization of the insulinoma. CT and MRI are the accepted first-line and second-line investigations, respectively, while MRI can potentially supersede CT in the coming days due to its higher sensitivity. Endoscopic ultrasonography and arterial stimulation venous sampling are superior to CT and MRI in preoperative localization but are invasive [4]. Our patient was diagnosed based on the clinical evaluation, plasma glucose, insulin, C-peptide, calcium, and parathyroid hormone level as well as imaging studies such as an ultrasound of the abdomen and neck, CECT abdomen, and ^99m^Tc MIBI scan.

There are two major challenges in managing patients with malignant insulinoma. First is the tumor itself, which is metastatic, and second is severe hypoglycemia [6]. Identification of patients at risk of aggressive disease is a challenge in treatment [8]. Patients have characteristic inappropriately elevated levels of insulin and proinsulin levels at diagnosis [6], but it does not differentiate between a benign and malignant or less and more aggressive form of the disease [7, 8]. An insulinoma is considered malignant when there is a local invasion to the surrounding tissue or lymph node or liver metastasis [11]. The lymph node or liver are the most common sites of metastasis in malignant insulinoma [4, 7]. Regional lymph node involvement is not always linked to poor prognosis [8]. Similarly, liver metastasis is commonly but not always associated with poor prognosis [7]. There are no predictive factors to determine the aggressive nature of malignant insulinoma and its propensity to metastasis, while only about 2% of benign insulinoma metastasize later [8]. Surgical removal of the tumor remains the mainstay of treatment of insulinoma, which reverts the neurological sequelae of hypoglycemia [4, 7]. Aggressive surgical resection favors survival and quality of life [4]. Neuroendocrine tumors are the histologic findings from both primary and metastatic sites [7]. Our patient underwent classical pancreaticoduodenectomy without any further episodes of hypoglycemia then after. HPE of the resected pancreatic lesion showed a well-differentiated neuroendocrine tumor with a single lymph node involvement and thus confirmed malignant insulinoma in our patient.

Primary hyperparathyroidism is associated with 90% of MEN1 which presents with hypercalcemia in 100% patients [1]. A parathyroid lesion in MEN1 is almost always (99%) benign adenoma or hyperplasia, while malignant parathyroid tumors are extremely rare [1]. Surgery is the preferred and most effective treatment. The timing of surgery depends on age, the severity of symptoms, PTH and calcium levels, and MEN1-associated endocrinopathies. [1] Our patient went for hemiparathyroidectomy a year later. In MEN1, besides tumors originating from parathyroid glands, endocrine gastroenteropancreatic tract, and anterior pituitary, nonfunctional adrenal and incidental thyroid lesions are found in 30–40% [12] and 25% [13], respectively. Hormonal and imaging studies revealed incidental nonfunctional adrenal and thyroid hyperplasias in our patient.

MEN1 presents with varied clinical features that warrant genetic testing for diagnosis in suspected cases and screen the family members for risk of development of the disease [14]. For mutation-positive individuals, focused surveillance for early identification of potentially malignant neuroendocrine tumors is recommended [1]. MEN1 carriers are recommended to be screened every 1–3 years for hyperparathyroidism, and other tumors as the clinical features are often mild for a longer duration [15]. A history of four undiagnosed deaths of her sons and brothers at early ages raises a strong suspicion of genetic association, but unfortunately, genetic testing was not done due to financial constraints.

## 4. Conclusions

Patients presenting with Whipple's triad should be further investigated with a high degree of suspicion for insulinoma. Eventhough MEN1 is a rare entity, a vigilant clinical approach to identify subtle clinical features of hypoglycemia along with localization and surgical removal of the resectable mass is imperative for treatment. Patients with an endocrine tumor should be investigated for the presence of other endocrine and/or neuroendocrine tumor(s) or syndrome.

## Data Availability

The data used to support the findings of this study are included within the article.
